# A home for body and soul: Substance using women in recovery

**DOI:** 10.1186/1477-7517-10-39

**Published:** 2013-12-20

**Authors:** Edward Kruk, Kathryn Sandberg

**Affiliations:** 1 The University of British Columbia, School of Social Work, 2080 West Mall, Vancouver, BC V6T 1Z2, Canada; 2 B.C. Ministry of Children and Family Development, Child and Youth Mental Health, Victoria and Campbell River, B.C., 1234 Gardener Way, Comox, BC V9M 4H5, Canada

## Abstract

**Background:**

We report on an in-depth qualitative study of 28 active and former substance addicted women of low or marginal income on the core components of a harm reduction-based addiction recovery program. These women volunteered to be interviewed about their perceptions of their therapeutic needs in their transition from substance addiction to recovery.

**Method:**

Data were gathered about women’s experiences and essential needs in addiction recovery, what helped and what hindered their past efforts in recovery, and their views of what would constitute an effective woman-centred recovery program. The research was based upon the experience and knowledge of the women in interaction with their communities and with recovery programs. The study was informed by harm reduction practice principles that emphasize the importance of individual experience in knowledge construction, reduction of harm, low threshold access, and the development of a hierarchy of needs in regard to addiction recovery.

**Results:**

Three core needs were identified by study participants: normalization and structure, biopsychosocial-spiritual safety, and social connection. What hindered recovery efforts as identified by participants was an inner urban location, prescriptive recovery, invidious treatment, lack of safety, distress-derived distraction, problem-focused treatment, coercive elements of mutual support groups, and social marginalization. What helped included connection in counselling and therapy, multidisciplinary service provision, spirituality focus, opportunities for learning and work, and a safe and flexible structure. Core components of an effective recovery program identified by women themselves stand in contrast to the views of service providers and policymakers, particularly in regard to the need for a rural location for residential programs, low threshold access, multidisciplinary service provision of conventional and complementary modalities and therapies for integrated healing, long-term multi-phase recovery, and variety and choice of programming.

**Conclusion:**

A key barrier to the addiction recovery of women is the present framework of addiction treatment, as well as current drug laws, policies and service delivery systems. The expectation of women is that harm reduction-based recovery services will facilitate safe, supportive transitioning from the point of the decision to access services, through independent living with community integration.

## Introduction

This article presents the results of a qualitative research project examining the perspective of 28 low income substance using women on the core components of a harm reduction-based addiction recovery program. In-depth interviews were completed with women living in Vancouver’s Downtown Eastside neighborhood, an area with the lowest per capita income and highest level of problem substance use in Canada, yet also at the vanguard of harm reduction programming on a global level.

The goals of the study were to learn about the primary addiction recovery needs and other core issues of women in their struggle with substance addiction (including alcohol, heroin, methadone, cocaine and crack cocaine, and metamphetamines), and to establish priorities for the development of a harm reduction-based addiction recovery program from the perspective of the women themselves. This data may be used to inform and guide policymakers, program developers and service providers in the development of such programs.

When we speak of “harm reduction” as a foundation to addiction recovery, we refer to a user-directed approach focused on the reduction of substance use-related harm without requiring abstinence from substances. This does not preclude abstinence for those women struggling with addiction who choose this option.

In this article we join the pillars of harm reduction and addiction recovery (including residential addiction treatment), and concur with the women who participated in our study that women seeking recovery from “hard drugs” (alcohol, heroin, methadone, cocaine and crack cocaine, and metamphetamines) should have a menu of choices made available to them in their recovery journey, including the options of both non-abstinence and abstinence-based recovery. In the opinion of the women we interviewed, abstinence should not be required for entry into recovery programs, as low threshold access is essential; abstinence was, however, seen to be to be a key element in ensuring women’s safety within residential recovery programs. Thus our definition of harm reduction also includes the option of abstinence-based recovery for women who choose it.

### Existing service provision

The most common services currently available to women struggling with substance addiction include detoxification, residential intensive treatment, residential supported recovery/community reintegration facilities, outpatient services, and mutual-aid groups [1-3]. Detoxification services typically involve short-term stays up to a maximum of two weeks, while the length of stay in intensive treatment programs may range from 30-90 days with typical stays of 4-6 weeks. Residential supported recovery facilities typically permit longer stays and some are open-ended. Many of the services are faith-based in Christian traditions and a significant majority utilizes the 12-Step method of recovery requiring attendance in Alcoholics Anonymous or Narcotics Anonymous meetings. Some programs do not accept clients who are on methadone maintenance and few have facilities to support parents with children. Typical programs include alcohol and drug focused counselling, relapse prevention and life skills sessions [4].

Trends in addiction recovery over the past decade include approaches that are harm reduction-based; that is, strength-based, client-centred, and respectful of self-determination and individuality [5,6], in place of pathologizing, and prescriptive, problem-focused, confrontational, “one-size-fits-all” approaches such as disease model and deficit-based “step” approaches that emphasize powerlessness over addiction and where treatment is delivered in a more directive fashion [7]. There is an increasing focus on holistic programming that may include body-mind therapies [8,9].

Although trends are slowly changing due to an increase in woman-focused research, the majority of current treatment supports and services are still misinformed by research with a “male-as-norm” bias [1,2,10,11]. As Neysmith [12] writes, “Change is hampered by the discourse available to us, which is concerned with the relations of power and control in our society as they are embedded in our policy documents, service structure, and professional practices”. This project provided an opportunity for women to engage in the addiction recovery discourse so that they may “make visible the realities of their daily lives and claim authority to shape their futures” ([13]: 185).

## Context of study: Vancouver downtown eastside

Estimates reveal a total of 5,000 active intravenous drug users (IDU’s) in the Downtown Eastside (DES) area of Vancouver [14,15]. In 1997, in response to the high rates of drug overdose and HIV transmission in the DES, a public health emergency was declared. The emergency remains acute for women in particular, as HIV incidence rates among female IDU’s in Vancouver are 40% higher than those of male IDU’s (ibid.; [16]). The primary response to the health and social emergency has been the creation of the “Four Pillar” approach comprised of prevention, treatment, harm reduction and enforcement, although intensifying law enforcement has remained the dominant approach [14]. Criminalization of drug users, however, is seen as a major factor fuelling the existing HIV and Hepatitis C epidemics. Law enforcement efforts include arrest and criminal justice involvement, with “tagging” of women involved in sex work, undercover sting operations, police harassment, and crackdowns. Sex workers’ use of “spotters” in the interests of their own safety are compromised by fear and distrust of police, and exiting the “score and use” lifestyle becomes difficult under these conditions [15].

Also fuelling the epidemic are inadequate detoxification facilities, lack of treatment facilities, deinstitutionalization of the mentally ill with inadequate community supports, the increasing geographical concentration of IDU’s in Vancouver, inadequate housing, the lower use of the supervised injection site by women as compared to men, and the lack of availability of heroin prescription for long-term users. Poverty is the overriding concern of women struggling with drug addiction in the DES, as social assistance payments are inadequate and welfare ineligibility is a core concern for many women. Low-income female drug users are regarded as “suspect tenants” and homelessness remains an issue for many. Women are routinely exposed to violence, and health issues include communicable disease, mental heath challenges, and lack of accessible health care (ibid.; [16]).

The relationship between drug addiction and sex work is strong among women in the DES. Twenty of the 28 women in our sample, for example, had been involved in long-term sex work. The cost of drugs has a profound effect on the frequency of sex work, because as prices rise, women become more desperate to earn income and competition for dates increases. Exposure to violence and increases in the rate of sexually transmitted disease also rises [15,17].

## Women and addiction recovery

The history of women’s addiction treatment and recovery reveals some interesting patterns, including the fact that women’s dependence on drug-abusing men has long been a key factor in their addiction [18]. Women’s addiction to alcohol and drugs has been largely iatrogenic, as women’s dependence on doctors has been a key factor in their addiction; this is evident in patterns of prescription of alcohol for women (common in the 1800’s); opium (1800’s); cocaine (late 1800’s/early 1900’s); barbiturates (1950’s onward); amphetamines, tranquilizers and sedatives (1960’s onward); and anti-depressant drugs today. Further, women’s addiction has often been the unintended outcome of well-meaning treatment approaches: morphine as a cure for alcoholism, cocaine for morphine, heroin for cocaine, methadone for heroin addiction.

From the 1800’s until today, women have been seen as needing longer treatment than men, and mostly addiction treatment for women has been an offshoot of men’s treatment, including Alcoholics Anonymous, as well as inebriate asylums (in the 1800’s); psychiatric asylums and sanitoriums (late 1800’s/early 1900’s); criminal justice-based treatment centres and psychiatric wards (mid-1900’s); and in-patient treatment and rehabilitation units (from the 1960s inwards).

It is now recognized that women have different addiction recovery needs than men, and that recovery processes for women should be gender- or women-sensitive to address their unique needs [1-4,19-26]: in general, women report more problems related to health and mental health, as well as more past trauma and abuse (physical and sexual), and experience more sexual problems. Women are more likely to begin using drugs after a specific traumatic event, and to suffer from post-traumatic stress disorder. Women experience higher levels of affective disorders, shame, depression, and anxiety (ibid). Importantly, women suffer from “low expectations,” as basic survival concerns dominate:

In general, chemically dependent women have been found to have lower expectations for their lives than male addicts, and they express greater preoccupation with simply surviving and minimizing discomfort than getting ahead in life [2].

As a group, women struggling with drug addiction have less education, fewer marketable skills, fewer work experiences, and diminished financial prospects compared to substance dependent men [27].

Most chemically dependent women who enter treatment are unemployed and have not been employed within the preceding year [15].

Chemically dependent women are more likely than men to be dependent on a family member or on public assistance for survival, yet they are also more likely to be primary caretakers of children [24,26,28-30].

Prescription drugs are more abused by women [25,26]. Women are more likely to use in isolation and in private (ibid). Women report greater concerns about children and child custody [27]. Women alter alcohol and drug intake with gender-specific biological events such as pregnancy, and intervention is more effective at pregnancy and childbirth. Longer-term treatment is more successful for women, as are post-treatment social support services [3,18,19,21].

Women are underrepresented in alcohol and drug recovery programs [19], and not only in regard to entry into programs; retention and completion rates are lower for women. Social stigma is a factor in this regard, as is the care of dependent children, the opposition of family and friends, economic barriers, with mistrust of service providers being more pronounced for women, and the fact that prescriptive and confrontational approaches have been largely ineffective with women (ibid). Goldberg [23] found that many recovery programs are unable to accommodate the needs of substance-abusing women and hence exclude them.

Beckman [19] developed a framework for understanding barriers to women’s successful recovery from substance addiction, distinguishing between internal and external constraints. Internal barriers include denial and minimization, fear of stigmatization, concern about leaving or losing children, and guilt and shame [29-35]. External barriers include interpersonal (the most significant being opposition by family and friends), and structural factors (including lack of economic resources, gender-specific services, and inadequate training of professionals). Beckman found that it is less common for women to seek services for substance use specifically or directly. The reasons most commonly given are depression and anxiety, medical problems related to use, and problems with partner, spouse or children. She concluded that, “the most critical aspect of treatment for women lies in therapists’ attitudes toward women” [19].

Other studies have stressed that women’s addictions are frequently correlated to issues of trauma including a history of repetitive childhood physical and/or sexual assault including incest and rape [21,23,36,37]. Women often use substances to cope with the pain associated with abuse at the hands of family members and partners, and users are subjected to more violence by their partners than are non-users (ibid; [38]).

Covington [1] proposed an integrated approach to women’s addiction recovery based on six principles: development and use of all-female groups in the stages of early recovery; recognition and address of multiple issues through a comprehensive, integrated, collaborative system of care; provision of an environmental setting that provides and promotes safety, respect and dignity; variety and choice of therapeutic approaches; a positive focus on women’s strengths and competencies; and individualized and matched treatment services. Russel & Gockel [39] identified four processes considered essential for women in recovery: validating the importance of relationships; providing mutual support; offering choices and multiple paths to healing; and eliminating punitive or confrontational strategies. Other key principles in women’s recovery include the need to address women’s fear of losing their children [1]; the need to maintain mothers’ parental role [27]; the importance of addressing women’s experiences of trauma and the physical, emotional and sexual abuse of women, and histories of incest and rape [1]. Addiction recovery for women is highly correlated with treatment for trauma, as trauma survivors often turn to drugs to medicate pain (a trigger for relapse). Women’s primary need is thus seeking safety and self-care in a supportive, structured environment (Herman, 1992) [40].

Other important principles related to the recovery of women struggling with drug addiction include:

-Recognizing drug and alcohol use as a coping response [1,6]

-Promoting awareness of and actively countering women’s oppression; taking into account gender roles and female socialization (i.e., not supporting passive, dependent roles) [19,20,29]

-Linking recovery with physical and spiritual transformation, and encouraging a richer spiritual life [1,31,41]

-Replacing addiction with a healthy lifestyle, an expanded self-concept, and focusing on healthy relationships ([17,21]; Herman, 1992 [40])

In sum, the literature suggests that the most important elements in women’s addiction treatment are: to regard the individual woman first (her needs and capacities) and then examine the problem and situation; avoid the tendency to label or define women as “addicts”—rather, see women as individuals with strengths and capacities; recognize heterogeneity among women and use individualized recovery plans; and match recovery to different subgroups (e.g., pregnant women, women with and without children). In addition, it is important to focus on physical, psychological and emotional, social, and spiritual needs, including safety and violence concerns, physical and mental health, housing and homelessness, financial and vocational needs, family and relationship challenges (and social connections and support), and basic life skills.

## Method

The study sought to provide an enumeration of the core needs of women struggling with drug addiction, including those related to addiction recovery, and to establish priorities for the development of a harm reduction-based women’s recovery program, from the perspective of women themselves. The views of low-income women in particular are lacking. Thus we designed an exploratory and descriptive qualitative study that utilized narrative inquiry (an examination and analysis of stories) as the main approach to data collection. It is rooted in grounded theory that leads to the discovery or generation of theory derived directly from the data provided by the stories, and informed and guided by harm reduction practice principles that emphasize the importance of individual experience in knowledge construction. It adopts a biopsychosocial-spiritual – or holistic – approach to understanding women’s needs, which postulates that substance misuse is the net result of a complex interaction between a combination of biological, psychological, social, environmental and spiritual determinants. We draw on May’s [42] definition of addiction as “a state of compulsion, obsession, or preoccupation that enslaves a person’s will and desire”.

### Sampling

The project received approval from the Behavioural Research Ethics Board at the University of British Columbia. Low-income women with substance addiction problems were purposively recruited and selected via posters placed variously in the DES neighborhood, in medical clinics and drug user associations, and with outreach and addiction-related service providers. The posters announced the focus of the project as seeking the input of women on core components of an effective addiction recovery program. Potential participants made initial contact directly by collect telephone call or indirectly through a service provider. The goals of the project were then explained, and consent form provided. No financial remuneration was offered to elicit participation. Every participant indicated that her decision to contribute to the study was driven by a desire to influence the direction and development of recovery programs that would effectively meet her needs and the needs of other women in the future.

### Data collection

Details of the study and the legal limits of confidentiality were explicated to all parties. Participants were informed that participation was voluntary and that the decision whether or not to participate would be known only to the investigators and was separate from, and would in no way jeopardize their participation in, any programs or services with which they may have been involved.

Each woman participated in one audio-taped face-to-face interview of approximately one hour in duration. Methodological instrumentation included two phases that consisted of narrative storytelling about each woman’s personal history as it relates to addiction, and a semi-structured interview with open-ended questions pertaining to needs, what has helped and what has hindered in meeting needs, and the principal components of a harm reduction-based addiction recovery program. The stories of the first phase were invited to provide context, and to assist the participants in accessing the memories that would help to inform their responses to the questions in the second phase.

### Data analysis

The research data - consisting of audio-recordings, their transcriptions and a field journal of ongoing notes - was approached using elements of a reflexive grounded theory approach of a constant comparative method and content analysis. This multi-modal approach involved switching between deductive and inductive reasoning and contextualization and decontextualization throughout analysis:

[Grounded theory] …*is not a specific technique, but rather a methodological orientation that seeks to base theorizing in the data rather than imposing a pre-determined hypothesis…a reflexive stance requires acknowledgement of the knowledge the researcher inevitably brings to the data…Such an approach involves examining the data with existing influences made explicit, but with an openness to the theoretical implications of the raw data.* ([43], p. 64).

In this study, a single-case analysis was conducted with each transcript. The classification system of Lieblich et al. [44] was used, including holistic-form, holistic-content, categorical-content and categorical-form analysis. The holistic-form approach examined narratives for turning points related to substance use and recovery. The holistic-content method was used to discover patterns and themes within the context of the whole story. Categorical-content analysis entailed the selection of subtext that corresponded to categories derived from the interview questions. Lastly, a cross-study analysis sought common themes and negative evidence.

Coding occurred at three levels: open coding (as data were collected, concepts were suggested or gathered implicitly by the original words of the participants); axial coding (as data were compared with new data, clusters of data emerged and categories were constructed and coded); and selective coding (integrating and refining of categories). To begin, each interview was read several times by the two researchers, with patterns summarized and exceptions, complexities and contradictions noted. The next step for each interview was to code the data into text units that represented a single “thought” or meaning. Each “thought” was numbered in consecutive order on the main body of the text, with labeled “thoughts” remaining close to the original language. The meaning units were then reviewed and a list of key words was made. If the words seemed to be important to the participant’s meaning and were not isolated references, the researchers considered whether or not they could be a part of a theme within that transcript. Key words were grouped and regrouped with other key words.

Discovering themes required more in-depth breakdown of the data. Each transcript was broken down into parts of various foci and the meaning units relating to each focus were listed. The meaning units were then examined to see what specifically occurred in each woman’s experience. The core themes in each transcript were identified by breaking down the data in this manner.

The next step was to create a theme matrix using cross-study analysis. All themes were listed and checked to see which ones were common across the transcripts; patterns and relationships between patterns were identified through constant comparative analyses among cases. Some of the final themes were of different wording than the originals; the wording of themes was changed to clarify, avoid repetition, or create an umbrella term that connected a number of sub-themes.

After studying the theme matrix, recontextualizing and attempting to discredit as well as link together the themes and potential metathemes, the final, higher-order themes were identified. Categorical-content analysis entailed the selection of subtext that corresponded to categories derived from the interview questions. This multi-modal approach involved switching between deductive and inductive reasoning and contextualization and decontextualization throughout the analysis.

### Research participants

Participants included 28 women who were either active or former users of “hard drugs” (alcohol, heroin, cocaine and crack cocaine, and metamphetamines) who self-identified as being of low or marginal income, and as being residents of Vancouver’s Downtown East Side. Both women in active use and in various stages of recovery were included in the study in order to ascertain which aspects of addiction recovery hindered or supported them in meeting their goals, and in recognition of the fact that substance use and recovery occur along a continuum, with constant changes in direction along the continuum. The women ranged in age from 22 to 59, with a mean age of 41. Half of the women were living in the DES or were homeless, while the remaining women had previously lived in the DES or were previously homeless. Five of the women identified as being of First Nations heritage, and 24 of the 28 participants were mothers. Five identified alcohol as their drug of choice and all of these identified as having a severe co-occurring mental health illness. Ten of the women were currently clean of drugs, five were using prescribed methadone only, eight were actively using drugs excluding methadone, and five were actively using drugs in addition to methadone. Five of the ten women who were clean had been so for a minimum of 6 months; two of these were clean for eight months, one for 9 months and two for several years.

Thus the majority of our participants were still actively using alcohol, heroin, cocaine, crack cocaine, and metamphetamines. Most did not have long-term sobriety. All but one of the women had experiences with one or more addiction recovery programs including inpatient and outpatient detoxification, residential treatment, day treatment programs, mutual support groups and programs within correctional facilities.

Pseudonyms were used for each participant to protect anonymity, which were chosen by the participants themselves.

### Limitations

The majority of the study participants were women for whom addiction services and programs had not proven helpful in overcoming addiction. Recruitment did not attract women with long-term sobriety who were completely satisfied with services. It seems likely that those women for whom services were helpful were not sufficiently motivated to volunteer as participants. This study attracted women who wanted to see changes in services.

More than half of the women in the study were actively using drugs, and were or had been both severely addicted and socially marginalized, homeless and engaged in illicit activities. As such, they are not representative of all women with addictions. However, the intention of this qualitative study was to learn more about the needs and experiences of the most socially marginalized women who struggle with substance addiction. Due to the highly personal and individualized nature of addiction, this data may also not be indicative of the experience of every woman of marginal income with addiction problems, but there is nothing to indicate that the most salient features of the results are unique to this sample. The commonalities and parallel experiences between the respondents support the conclusion that they are representative of the population in question.

Due to the lifestyles and situation of the majority of the women, the researchers were unable to conduct member checks.

## Results

Our findings are organized into the following sections: recovery needs, what hinders recovery, what helps, and core components of an effective harm reduction-based recovery program.

### Needs

Women’s self-identified primary needs vis-à-vis addiction recovery were as follows, in order of importance: normalization, the need for residential recovery facilities, an open door, biopsychosocial-spiritual safety, time, ongoing support, a home, and financial assistance.

#### Normalization

The most fundamental need of women struggling with drug addiction is to “be normal”. Within the context of our findings the word denotes a state of being that is free of the “slavery” and affliction of drug addiction. The women reflected an ongoing preoccupation with thoughts around becoming “normal,” even while they were actively using substances:

*I always used to look out the window and see people and wonder why they’re laughing, jumping, skipping rope, and throwing balls to their kids, and - you know - wondering, “Why God? Why don’t I? Why am I not like that?”… I just wanted to be a human being… A normal human being.* (Susan).

#### A recovery facility

All except one woman indicated a need for more residential recovery facilities. An unexpected finding was that 26 of the 28 women felt that such facilities need to be rurally-based, well away from the atmosphere of the DES and the urban environment, and that a core component of harm reduction-based recovery was the healing afforded amidst nature within a rural setting.

Every woman in the study indicated that she wanted women-only group work in residential programming, and some preferred a women-only residence. Leigh said, “I think it should be strictly women. I think there’s a huge part in a woman addict helping another woman addict…I just think it’s amazing. It’s like a miracle. Magic happens…” Others indicated that some family or couples group counselling would also be beneficial in the later stages of recovery.

Participants without children who lived with life partners wanted to see more residential centers for couples, and participants currently living with minor children wanted residential facilities that could accommodate the entire family. Participants with adult children or with children living permanently in care did not indicate whether or not a family inclusive centre would have been of benefit to them in the past.

Throughout the interviews the women expressed a profound need for time to focus on oneself without distraction, and that the capacity to focus on self could be complicated by familial and marital concerns, including the pain of separation itself. Women fear for their children’s safety when they are placed in temporary foster care for the duration of an addiction recovery program, and are afraid that their children may be permanently removed. However, women also recognized the need for some separation. The women interviewed wanted separate living quarters from their spouses in the stages of early recovery, and while they would like access to their children, they wanted the option to be apart from their children. They did not want to assume full responsibility for their children’s care. Some suggested that child care be provided within the facility according to the needs of the mother; others wanted regular contact with their children, with their spouses or other family members assuming responsibility for child care.

A number of women expressed ambivalence around the issue of life partners in recovery together when both parties are struggling with addiction. They acknowledged that they each had the capacity to both help and hinder the other’s progress.

#### An open door

*If I want to stay clean, I have to be able to walk through that door right now, push that button and say, “I’m high. I have no drugs on me. I need a bath and I want to get clean.” And not turned away…[I need] recovery on demand. That means that I don’t have to be clean for 72 hours or 48 hours – even 24 hours. Because when you turn somebody down that’s dope sick, and say “No. We won’t help you stay clean”, I’m going to the dope dealer.* (Marina).

The need and inability to access detoxification and recovery programs upon demand were consistently topics of passionate and animated discussion. The women recounted numerous experiences of repeated and frequently desperate attempts to gain access to services that failed because of various admission criteria or lack of space. Another identified barrier to access was the daunting process of navigation to the location of the service when the route is wrought with difficulties, pitfalls, temptations, and triggers of many forms, including, but not limited to, poor health, dope sickness, cravings, drugs, drug pushers, using friends, and lack of money, transportation and energy. For women living in the DES, such navigational difficulties are extreme. They may find it impossible to leave a hotel room without immediately encountering drugs in the hallway, and will most certainly encounter them on the street.

*There’s so many barriers, you know, so many barriers…that some things have to be made easy*. (Joni).

#### *Biopsychosocial-spiritual and environmental safety*

Addiction treatment centers had not proven to be safe places of recovery for many of the women. Drugs were sometimes smuggled in by clients or visitors and the women indicated that they had been subject to violations in varying degrees from both clients and staff that included manipulation, indoctrination, coercion, and physical, mental, and sexual abuse and exploitation.

[We need] a safe place where there is no use, where there is no abuse, where there’s no one trying to hurt us…change our minds…someone who’s there to see us heal physically, mentally and spiritually…

*Someone who’s there to promote our health with rules. I have total respect for rules, however…not someone who’s trying to push a religion on me, or a belief system. Someone who may want me to try that system and that I will try with an open mind, but not when someone cuts down every other method.* (Athena).

Trust was a major issue for women in recovery programs and some women found it extremely difficult to open up in a group. Some women suggested that intensive group work should not commence until trust has been established. This may require time and/or it may require a specific trust-building component to be structured into the programming.

*I think being really, really clear and having a definite process of trying to build trust among a group of people…I’m not sure exactly what you do to build trust but it has to be explicit. It has to be a primary focus because you’re not just building trust with those people who are in the group with you, you’re ultimately building trust in the other people you encounter in your life*. (Joni).

#### Time

The women talked about needing time for their recovery process. They need time to wean off drugs, to come “back down to earth,” to stabilize, for their “brains to recover,” to think straight, to build back up, to learn to trust, to learn to live in new worlds, to develop a new lifestyle, to reintegrate into the workforce, to work on life issues, to reach a place where triggers are not overwhelming, to move from chaos into peace, and to replace old experiences with new. While the majority of participants emphasized that stays in detoxification and residential centers should have flexible timelines according to the unique needs of the individual, and that access to aftercare should be open and ongoing as needed, it was frequently suggested that a two-year period could be a viable time frame in which to realize many of these goals.

*We are disrupting a whole history…* (Joni).

#### Ongoing support

Ongoing support refers to any and all physical, psychological, social and spiritual supports that the women find helpful in the maintenance of their well-being after they leave a residential center. These include, but are not limited to, professional aftercare services, mutual support groups, and natural supports such as family and friends.

*I would say my biggest need right now is support. Just to know that if I needed somebody to be there that they would be. I think it’s more empowering to know that there’s groups that you can go to, people that you can talk to, and when situations get a little out of control you don’t have to turn to a drug - that there’s somebody there who would listen to you.* (Dee-Dee).

*Once you’re on your own, even if you’ve gotten yourself an apartment and if you’ve gotten yourself a job - to be able to keep going back - maybe not to stay and reside, but if you need to go back and talk about relapse or whatnot*. (Grace).

#### A home

The women say they need “homes,” as opposed to merely “housing.” There is a qualitative difference between the two that the women made clear using descriptors like ‘safe’, ‘warm’, and ‘comfortable,’ to describe the former. They need homes from which to successfully access recovery programs, and in which they can stabilize after a residential program.

*We move out [of residential programs]. We’re clean; sober; we don’t have any money. We get stuck with roommates and they’re using. We get stuck in shit holes ‘cause we don’t have enough money. Low income [housing] is synonymous with crack shacks. You’re living in places of squalor and drug addiction. So if we had places subsidized and really strictly no using, no partying, no this, no that…you’d have maybe a better chance. Because I don’t care - you can be as strong as you want – if you’ve got somebody doing an eight ball in your living room, it’s not conducive to staying straight*. (Athena).

#### Financial assistance

Women need assurances that their rent, bills and other financial obligations will be provided for while they are in recovery programs, and provisions to reestablish themselves within communities afterwards. “…I wasn’t just giving up a drug. I was giving up a kind of income.” (Joni).

### What hindered recovery

What hindered successful recovery in the women’s experience were: an inner urban location, prescriptive recovery, invidious treatment, lack of safety, distress-derived distraction, problem-focused treatment, aspects of mutual support groups, and social marginalization.

#### Inner urban location

Locating recovery programs and residential centers in areas where drug users congregate was considered absurd by the respondents, due to excessive exposure to triggers and access to drugs. One participant described her reaction to the surroundings of a treatment facility upon her arrival as follows:

*At first I was like “Excuse me? Are you sure?” I’ve sat on that corner before and I wasn’t in treatment – that’s for damn sure. So I asked about it. “What the hell are you guys thinking making a treatment center right here? I mean, there are crack-heads in the alley and drunks everywhere, and drug dealers everywhere”. They explained to me that the reason they chose that location was because that’s where they got the funding for it…I just found it kind of stupid to be there*. (Leigh).

#### Prescriptive recovery

Prescriptive recovery may be understood as a recovery method that is imposed, enforced or sanctioned with exact rules, directives or instructions about how it must occur. A major theme throughout the interviews was the dehumanizing effect of prescriptive recovery with its disregard for individual difference and self-determination. Jewel complained of expectations that she be “just like a robot”. The word “robot” appeared in several interviews in reference to such an approach. Ginger was representative of the women when she stated, “Everybody’s recovery is going to be different, you know. Different things work for some people that don’t work for other people.”

#### Invidious treatment

Past experiences of invidious treatment by caregiving professionals was reported as negatively impacting desire and decisions to seek help for addiction. Deficit-based and confrontational methods of counselling, judgmental attitudes, omniscient demeanors, and “pushy” behaviors are barriers to successful engagement and retention in addiction recovery programs.

#### Lack of safety

A description of safety issues and corresponding harms are discussed in the above section on “Needs,” under the sub-heading “Biopschosocial-spiritual and Environmental Safety”.

#### Distress-derived distraction

Personal anguish or suffering can interfere with women’s abilities to focus on their personal needs in the change process and hence impede progress. Such anguish cited included boredom, depression, general anxiety, low self-esteem, fear, and interpersonal concerns (especially with spouses and children). Several women recommended that telephone calls and visitation be strictly limited or prohibited during the early phases of recovery to shelter them from outside concerns, and thus support them in focusing on their own recovery needs.

#### Problem-focused treatment

Problem-focused treatment, with an emphasis on problems, deficits, weakness, illness and even an over-emphasis on substance use, may prove debilitating and ultimately defeating. Treatment or supports in which personal accounts of addiction struggles and “war stories” dominate discourse can be triggering and detrimental to a recovering substance user. The women overwhelmingly indicated a preference for a positive, strengths-based, and balanced approach to addiction recovery. “Life is supposed to be balanced. If it’s too one-sided – recovery, recovery, recovery – it’s too much for a person and it’s all about all my problems and my life history. It’s too much.” (Athena). Joni’s hard-won struggle with addiction taught her that, “To look beyond a substance is the way to be okay.” (Joni).

#### Aspects of mutual support groups

Nineteen of the women shared their experiences of step-based, mutual aid support groups, including Narcotics Anonymous (NA) and Alcoholics Anonymous (AA). While certain aspects of mutual support groups were considered helpful, every participant but two spoke emphatically about negative elements of AA or NA attendance, and of how such groups have the capacity to hinder as opposed to support women. Eight of the eleven women who had participated in NA found it more harmful than helpful while three found it to be helpful. Seven of the fourteen women who had participated in AA found it more harmful than helpful while five found it to be helpful overall, with two ambivalent about the experience. While two of the women were strong proponents of these programs, they also acknowledged dangers. Negative aspects cited included prescriptive methods of recovery including pressure to embrace each of the 12 Steps, the use of fear tactics and confrontation, a problem focus, unstable and unsafe members, judgmental attitudes, lack of confidentiality, war stories, emphasis on trusting a higher power when they found it difficult to trust at all, unhealthy romantic entanglements, deleterious attacks on self-esteem and increased exposure to drugs. Michelle described NA as “a social club for users.” Offence was taken by many women to AA and NA on the perceived grounds that the groups purported to be “the only way”; if one did not accept the tenets of the program she was often dismissed as “not ready” to seriously address her addiction.

#### Social marginalization

Marginalization can be understood as a process in which people who are relatively different from the dominant norm are moved to the periphery of society because of their identities, associations, experiences or environment. Women spoke in a desultory manner about “exclusion being a big part of addiction” (Michelle) and of being “treated like an outcast” (Melissa), or like “rats” (Athena; Michelle). Some of the women described how they were not permitted to sit in certain public areas or were singled out of crowds and harassed by police. “They don’t tell normal society to leave” (Grace). “Just because you’re a junkie doesn’t mean you’re a bad person” (Athena). The stigma and marginalization experiences of the women were linked to prevailing drug laws and policies which blame, stereotype and isolate low-income women struggling with illicit drug addiction in particular.

### What helped recovery

What helped women in their recovery process included: connection in counselling; multidisciplinary service provision; spirituality; keeping busy; an experience of exploration, learning, and discovery; work; a safe and flexible structure; nutritious food; and aspects of mutual support groups.

#### Connection in counselling

The women were unanimous in reporting that access to a peer or professional counsellor who had a history of addiction recovery was essential, and each First Nations woman stated that she felt more comfortable with a First Nations counsellor.

Counsellors with similar histories to the women are role models who represent hope and can share personally informed knowledge on how they were able to overcome their struggles and offer practical suggestions. The women reported being more likely to find them empathetic and compassionate, to trust them and to feel understood because of their commonalities. They appreciated the interpersonal insight that came from being “street-wise” and avoiding “bullshit” (Joni), or in other words, dishonesty in themselves or others. Such counsellors are favored over pedantic “textbook” (Athena; Rose) helpers. The women needed to “feel connected” to a counsellor.

*…there have been so many times when the very first person I’ll sit down and talk to, I don’t have that initial connection with. There seems to be a barrier…And yet the next time I’ll try again or go a different avenue and meet somebody completely different who I do connect with, and makes me feel like, “Yeah. I am a valuable person. I do belong here. I do need help, and you’re here, and I’m glad.”* (Dee-Dee).

Some of the adjectives used to describe the attributes of the people who helped the women were: “positive,” “professional,” “well-trained,” “compassionate,” “understanding,” “genuine,” “street-wise,” “warm,” “loving,” “tender,” “approachable,” “honest,” “respectful,” “accepting” and “welcoming.” Some appreciated a spiritually mindful counsellor who “knows how to bring you to that place - whatever you want to call it - closer to your higher power…” (Susan).

While most of the women appreciated group work in varying degrees, many indicated that they had a preference for one-on-one counselling, and several asserted that 24 hour access to a counsellor is critical. Some participants discussed the potential benefits of an informal approach to counselling that could occur while participating in ordinary activities like swimming, walking, and television viewing.

#### Multidisciplinary service provision

Most of the women in the study had complex needs and required multidisciplinary service provision. Several had physical disabilities and health problems, including HIV, AIDS, hepatitis C, diabetes and other serious illnesses. Several women were on medication for severe and persistent mental health issues like chronic depression, anxiety and bi-polar disorder.

*The main thing I needed to feel was that there was a team that was in control…for me to be able to let go and trust. There was a system in place.* (Sophie).

#### Spirituality

Spirituality (as opposed to dogmatic religiosity) was a major theme in the healing process. Leigh summed it up by saying, “I couldn’t understand how anyone could go through recovery and not have spirituality of some kind.” Spirituality presents in many forms while having an association with nature for a number of these women. Joanne described her own experience as follows.

I think I was always spiritual. I just kind of lost that in my life along the way. I’m getting that back and I feel more. I just don’t feel lonely all the time. My best friend was my bottle or whatever. Spirituality slowed me down, so I can at least focus. I can read now…and now can sleep. I don’t think I slept in four years…

I find my spirituality in myself, kind of, and just, I don’t know, like, whether you want to call it God or whatever. It’s almost like something’s embracing me. It’s everywhere – like the forest. Like, I go for walks and it’s absolutely beautiful in there and I take the girls and it’s just peaceful – which I’ve never had in my life – where I felt peaceful and safe. With those two things, I feel like I could sort of conquer everything. That and, with sobriety, being peaceful and safe.

First Nations women and Caucasian women both talked about the benefits of First Nations cultural teachings and ceremonies, such as sweat lodge and burning ceremonies. Other cited sources of spiritual benefit including 12-step and 16-step groups, and feminist spiritual teachings. Kasl’s [45] 16-step program is a feminist-informed, empowerment-based alternative to the more prescriptive approach of 12-step groups.

#### Keeping busy

Several women indicated that “keeping busy” alleviates preoccupation with drug use and facilitates the process of normalization.

*…not just keeping busy by going to group therapies and things like that. But keeping busy doing normal things…I really think trying to do normal things really, really helps and makes a difference because it gets you back into the track of what it’s like to live a normal life. And when you start doing stuff like that, you start to remember how much it actually was enjoyable. You know, when you get into this kind of lifestyle, you kind of forget how straight people actually have fun.* (Ginger).

#### Experience of exploration, learning, and discovery

The experience of exploration with learning and discovery was a key to the success of recovery for these participants. The following were identified by the women as essential:

* Body-mind exploration: walking; yoga; meditation; acupuncture; fitness/ recreational therapy; massage; tai-chi; self-hypnosis; touch therapies; expressive therapies; horticultural therapy.

* Self exploration: self-knowledge/honesty; trust building; grief and loss; physical, mental, sexual abuse; other trauma; relationships; self-esteem/self-empowerment; anger; thought management.

* Life exploration/education: drug and alcohol education (especially the personal impact of substance use); basic living skills; relationship skills; parenting skills; employment counselling; job training; alternative behaviors and activities; family and community reconnection and integration; spiritual paths.

Many of the women liked the idea of having healthy community members attend residential recovery programs to interact and give classes, workshops, lectures and to otherwise share of their time and knowledge, covering a wide range of topics.

#### Work

Work – whether it is volunteered or remunerated – was seen to provide stability, a meaningful role, a sense of belonging and increased self-esteem.

*…I like the routine of it…the getting up in the early morning with the sunrise, and getting your shower, and making your bed before breakfast, and helping and…doing the chores was phenomenal…I needed that…like I had been a really good housewife but I hated it. But this was different. This was me not having all the jobs but having a role. So I felt like I belonged. I was helping*. (Athena).

#### A safe and flexible structure

One of the dominant themes was that an effective recovery program must respect individual differences, and that the program structure must incorporate mechanisms that allow for self-determination. The women wanted a safe, flexible structure that has not only a set routine and clear rules, but many programming options. The women were adamant that while strict rules are necessary for safety, alternatives should be offered in programming, timelines should be dependent on needs and goals, and concessions need to be made for individuals, dependent upon their physical, mental, emotional, social, and spiritual needs and abilities. Brenda summed it up neatly, “Everybody is individual, you know, what works for one doesn’t work for another”. (Brenda).

#### Food

Nutritious, tasty food is essential to nurture the health and well-being of women who have been chemically addicted. Several mentioned that they would like access to food 24 hours a day in a residential center. Access is especially important in early recovery when they may be sick and unable to eat during scheduled mealtimes.

#### Aspects of mutual support groups

Some mutual support groups were found to be helpful to some for establishing a peer community with common bonds, as they provide accessible and low threshold access, attention to core needs including trauma support, and in some cases a spiritual pathway of recovery while providing support toward abstinence. In more empowerment-based and less prescriptive mutual aid groups, women found emotional support, respite from loneliness and an opportunity to learn from and with peers. Some found spiritually-based programming particularly helpful, and some of the women who had utilized support groups stated a preference for Kasl’s [45] 16-step addiction recovery model.

### Core components of an effective harm reduction-based recovery program

Our development of the core components of a harm reduction-based women’s recovery program draws on the wisdom and experiential knowledge of the participants in this study, and considers their stated recovery needs, and what has helped and hindered in meeting these needs in their past experience. Although the women did not use the term, “harm reduction” in their vision of the main components of an effective substance addiction recovery program, the components they identified are in fact core elements of a harm reduction approach. The women’s expectation is that recovery services include a residential component to facilitate safe, supportive transitioning from the point of the decision to access services, through independent living with community integration.

Every woman ardently asserted that residential recovery programs in particular should not be situated in areas that are frequented by people with addictions, or by drug dealers. With the exception of two women who preferred suburban residential neighborhoods, all stated that residential programs must be located in a rural location. For the vast majority of the women interviewed, the ideal location for a residential center would be in a quiet, safe rural area - preferably on an acreage - near water, trees and wilderness; away from cities’ dense population and traffic noise; far enough away to present as a barrier to an impulsive return to one’s former lifestyle, and a barrier to unwelcome individuals; close enough to a community to allow for weekly outings and community interaction; in a natural environment with walking areas and a garden. A number of women described a farm-like setting with animals.

*They always say you can’t run from your problems, or move from your problems - which is true - but taking a break doesn’t hurt either*. (Melissa).

*It’s just easier to clear your mind…* (Rose).

*…I think it’s partly that noise and cars and living on top of other people and dull roar…traffic and stuff in the background…kind of interferes with your own inner feeling or awareness. So, for me, being outside of this makes a huge difference. I mean I can hear myself and think and be open in a way that I can’t be otherwise…A lot of times in the city I’m so closed off just to minimize the battering feeling of all that, that it takes away from being able to be open to other things*. (Joni).

The following is the list of core components of a rural harm reduction-based women’s recovery facility as identified by the respondents:

* An overall approach that is holistic; respectful; positive; strengths-based; person-centred; supportive of self-determination and choice; balanced

* Low threshold access

* Safety (physically-psychosocially-spiritually and environmentally)

•Free from danger, including but not limited to, coercion, indoctrination, invasive and invidious contact and treatment

•Regulated

•Supervised

* Serene and home-like environment

* Multidisciplinary service provision of conventional and complementary modalities and therapies for integrated healing

* Long term multi-phase recovery program

* Flexible structure/timelines in consideration of individual need, ability and stage of growth

* Counselling readily available

•Therapeutic relationships

•One-on-one as needed

•Women-only group work in the early phases

•Non-confrontational approach and promotion of honesty

•Access to counsellors and support workers of similar culture and personal history (especially counsellors with an addiction history)

* Variety and choice of programming/therapies/activities

* An experience of physical-psychosocial-spiritual exploration, learning, discovery, and fun

•Body-mind therapies

•Sports, play and hobbies; self-defense training

•Personal self-development

•Spiritual paths

•Life skills

•Employment and educational counselling

•Work (volunteer and remunerated; supported as needed)

•Trust-building programs

•Drug and alcohol education

•Community interaction/integration

•Family counselling/education

* Connection with resources for ongoing support

•Supported homes

•Financial assistance

•Healthy mutual support groups

In regard to mutual aid groups, the participants in our study emphasized the importance of adopting a biopsychosocial-spiritual understanding of addiction, and a non-judgmental and non-hierarchical approach; a strengths and capacity-based approach emphasizing self-efficacy; low threshold access; social support; and the safety and structure of abstinence.

## Discussion and conclusion

From the perspective of low income women struggling with addiction, a harm reduction orientation is essential to the effectiveness of substance addiction recovery programs, and the two “pillars” of harm reduction and addiction treatment are inextricably linked. Although the women in our study did not necessarily use the term, “harm reduction-based” in their description of the core components of an addiction recovery program and center, the main elements they identified were first, the reduction of harm via attending to the basic needs of women in recovery (a needs hierarchy from their own perspective); and second, women-directed programs, both goals and strategies of a harm reduction approach [5,6,46]. Further, recognizing the continuum of drug use from non-problematic to problematic, the women emphatically emphasized the importance of low-threshold access to abstinence-based recovery programs (abstinence should never be a barrier to entry into recovery programs), another essential element of a harm reduction approach (ibid).

The women were forthright and keen to articulate their experiences and insights toward the goal of providing information that would make a positive and significant difference in the provision of what are essentially harm reduction-based recovery programs and services to marginalized women struggling with addiction. Our findings report women’s stated recovery needs, what hindered their recovery in past programs, what helped, and the core components of an effective harm reduction-based recovery program. These results not only complement existing theoretical and empirical formulations of the unique needs of women struggling with substance addiction, but add new data to our collective understanding of what is most salient to these women in their recovery process.

Central to an understanding of the core components of a model harm reduction-based recovery program is the adoption of a “kaleidoscopic” lens that does not view a constructed model as immutable, but understands that it provides a framework for systems of variation that reflect and respond to the complex and shifting states of the diverse entities it serves. Core concepts and principles comprise the girders of a framework and a guide to program activity, without assuming or insisting that every harm reduction-based recovery program based on these components be identical.

Our findings indicate that the three core needs identified by low income women struggling with drug addiction are normalization/structure, safety, and social connection. An effective recovery program must first and foremost address these needs, and a residential recovery program is essential for those who choose an abstinence-based approach, best located in a rural, natural setting. Recovery programs based on harm reduction principles need to have a safe, supportive, and flexible structure that responds to women’s’ unique needs and abilities, and supports individuality and self-determination. The intervention approaches used need to be positive, client-centered and strengths-based, as opposed to problem-focused, prescriptive, and deficit-based. They must provide a variety of biopsychosocial-spiritual experiences and education to assist the women in the discovery, development and/or remembrance of integral and core internal and external organizational constructs, processes, beliefs, behaviors and activities that will holistically facilitate the normalization of health, experience and lifestyle with personal and social integration. Such healing requires time, timeliness of intervention, and a safe, supportive and stable environment.

In keeping with a harm reduction orientation, the women in our study also identified the following key components of an effective addiction recovery program: immediate attention to reducing the harms associated with substance use and containing the adverse consequences of use; low threshold access to residential and out-patient services as needed; client-directed recovery tailored to the unique needs of each individual woman; a menu of choices and options in regard to intervention strategies, to maximize self-efficacy and develop a sense of mastery over what initially seem to be insurmountable obstacles. The recognition among service providers that there exists a continuum of substance use from non-problematic to problematic was seen as critical; when substance use becomes problematic to the degree that addiction is a concern, the need for long-term residential programs in particular was identified, and in the interests of women’s safety in residential addiction treatment, an abstinence requirement was seen to be fundamental [46]. From the women’s perspective, although abstinence should not be required for entry into addiction recovery, within a residential program it is a necessity for those who choose this option. This was in fact the preferred path of addiction recovery for the women we interviewed, with harm reduction programs seen as a gateway into abstinence programs. The implication for harm reduction practitioners is that for low-income women struggling with substance addiction, entry into addiction recovery programs should not require abstinence; however, once women make the decision to pursue abstinence in their process of recovery, they require the safety and structure of an abstinence-based residential recovery program. When abstinence is identified as the option of choice, this should be available within the menu of choices offered by harm reduction practitioners [5]. As an intervention, harm reduction is a program or policy directed toward decreasing the adverse health, social, and economic consequences of substance use without requiring abstinence, but it can include abstinence [46].

In sum, according to the women, the process of addiction recovery should direct attention to immediate needs, with women then co-constructing a hierarchy of their needs with the service provider, along with a menu of choices in regard to intervention, the provision of different interventions for women at different stages of addiction recovery, and service provider transparency in regard to the use of specific intervention strategies. These are essentially the core elements of harm reduction as a psychotherapeutic approach in addictions [5].

A notable finding is this study was that a residential recovery program should be located well away from an urban environment, where accessibility to drugs is a constant threat. A rural, nature-based residential setting was identified as crucial for addiction recovery by 26 of the 28 women. Women with substance use problems have talents, abilities and resiliencies that must be built upon so that they may empower themselves as self-effective agents of change, and a rural recovery setting would allow these strengths to re-emerge. Strengths-based practitioners in such a setting would look for and try to nurture the ‘gleam’ that is often hidden by misery, protective strategies, and the failure to achieve goals set by others [6].

People’s lives are lived through both subjective and objective transactions between inner and outer realities that are constantly developing processes rather than static structures (Linehan, 1993) [47]. A person is not an isolated individual but an ever-changing “self-social unity” (Lichtenberg & Roman, 1990) [48], both an object of the prevailing social order and a subject able to move beyond it [49]. The women in our study indicated that new experiences in and of themselves are transformational, so that the very experience of “normal” activity provides the means to normalize. New experience provides these women with a new storyline and new memories on which to build. They expressed a strong preference for body-mind therapies and activities, and indicated that they could best connect with and internalize emergent serenity and spirituality in a rural environment. These ideas are all supported by studies in psychophysiology that demonstrate the connections and interactions between the body, mind and environment [8]. The ideas we carry, habits we have, and society of which we are a part all interact with our biology [9]. Our habits continuously modify our brain function, and our brain function modifies both our existing habits and our abilities to acquire new learning and new habits (ibid.).

The assumption inherent in a biopsychosocial-spiritual – or holistic – approach to harm reduction-based recovery is that problems arise when individual characteristics combine with biological, psychological, social, environmental and spiritual factors to produce unwanted or harmful states and behaviors. The unique qualities of each individual in interaction with these factors and the unique manifestations of these interactions in the form of substance use and addiction construct a heterogeneous population of substance users with multi-varied needs and desires [21,50].

The complexities of the interactions that occur within unique ways in each individual demand a wide range of intervention options. There is no universal approach appropriate for everyone, and so it is desirable to match interventions with an individual’s characteristics, values, beliefs, strengths, needs and wishes within the context of her situation, and respecting social differences in race, class, gender, sexual orientation, religion, age, ability and ethnicity. Variety and options are critical and require harm reduction-based recovery programmers to be creative and resourceful in developing methods to offer a diverse group meaningful and effective experience. More interdisciplinary research should be conducted to develop a stronger knowledge base around body-mind science and the implications for addiction recovery. Drawing on this knowledge, policymakers will be in a better position to develop best practice guidelines that will meet the needs of women as efficiently and efficaciously as possible. Long-term and multidisciplinary service provision is essential in order to offer both conventional and complementary programming in relation to trauma and addiction ([3,21]; Gregoire & Snively, 2001 [51]; [1,36]; Herman, 1992 [40]).

Our research results also indicate a need for further examination and discussion of women’s experiences in AA and NA groups [46]. It is essential to seriously deliberate the further development of alternative support groups for women for whom the hindrances of these groups outweigh what helps. This issue is critical given the addiction recovery community’s reliance on AA and NA to provide community-based support services, and given women’s need for ongoing social support, safety and structure in the process of harm reduction-based recovery. The aspects of these programs that some women found unhelpful were the use of a prescribed rather than co-constructed approach to addiction recovery (to the extent that AA and NA follow a consultative model of co-direction of a recovery program they are beneficial); limitation rather than expansion of recovery options; and adherence to a “disease” theory of addiction (as opposed a strengths-based model using a “biopsychosocial” and “biopsychosocial-spiritual” understanding); and any interventions undermining women’s sense of mastery and control, as self-efficacy is a key element in addiction recovery [5].

Finally, the implications of our findings for programs developers and policymakers are profound. Although the feasibility of establishing the type of recovery programs envisioned by the women may be argued to be unrealistic and not feasible, it seems clear that an alternative approach to addiction recovery is urgently needed in the case of highly marginalized women struggling with addiction, who remain in constant danger of death and disease, and for whom existing recovery approaches have limited utility [15,16]. Long-term recovery [51], multidisciplinary approaches [1,3], and individualized recovery in residential care [3] are particularly important in women’s recovery, and the consistent emphasis on rural and nature-based facilities and holistic approaches among our respondents lead us to conclude that the inclusion of these components must be seriously considered by program developers and policymakers in the field of addiction recovery. Considering that rural areas have less expensive real estate options for residential treatment facilities, multidisciplinary teams do not have to reside at the facility or be accessible on a daily basis, and counselling may be offered by a wide range of non-clinical practitioners (lay and peer counsellors, massage and other therapeutic practitioners, mentors and spiritual leaders), such a recovery program may be more feasible than is commonly assumed. Finally, to the degree that they are able, the women themselves can work together towards the facility operating as self-sufficiently as possible, pursuing work projects with therapeutic elements such as growing a community garden, and those who have proven to be stable over time may pursue paid employment or work in exchange for goods and services within the community.

## Competing interest

The authors declare that they have no competing interests.

## Authors’ contributions

Both authors conceived of the study. EK designed the instrument for the study, and led the study’s design and coordination. KS carried out the interviews and qualitative data analysis. Both authors contributed to the drafting of the manuscript, and read and approved the final manuscript.
